# Misdiagnosis of Bland-White-Garland Syndrome: Report of Two Cases with Different Presentations

**DOI:** 10.5681/jcvtr.2014.013

**Published:** 2014-03-21

**Authors:** Akbar Molaei, Bahman Rastkar Hemmati, Hashem Khosroshahi, Madjid Malaki, Roya Zakeri

**Affiliations:** ^1^Cardiovascular Research Center, Tabriz University of Medical Sciences, Tabriz, Iran; ^2^Department of Paediatric Cardiology, Bozok University, Ankara, Turkey; ^3^Department of Tuberculosis and Pulmonary Diseases Center, Tabriz University of Medical Sciences, Tabriz, Iran; ^4^Tabriz Children Hospital, Tabriz University of Medical Sciences, Tabriz, Iran

**Keywords:** ALCAPA Syndrome, Dilated Cardiomyopathy, Prolapsed Mitral Valve

## Abstract

Anomalous origin of the left coronary artery from the pulmonary artery (ALCAPA) or Bland-White-Garland syndrome is usually an isolated cardiac anomaly but, in rare incidences, has been described with patent ductus arteriosus, ventricular septal defect, and tetralogy of Fallot. This syndrome may cause sudden death in infants and young people but in this case report we present two different types of presentation. First case was a 3 year old girl diagnosed with dilated cardiomyopathy since her infancy. Her electrocardiography showed prominent Q wave in lateral leads. Dilated right coronary artery was revealed by echocardiography. The second case was a girl with prolapsed mitral valve and chest pain but similar to first case she had prominent Q wave in lateral leads at her electrocardiography and dilated right coronary artery but without heart failure. ALCAPA in children may present with ambiguous presentations differing from dilated cardiomyopathy and full blown heart failure to an atypical chest pain attributed to prolapsed mitral valve.

## Introduction

Anomalous origin of the left coronary artery arising from the pulmonary artery (Bland-White-Garland syndrome also known as ALCAPA syndrome), is a rare congenital abnormality affecting 1 in 300.000 live births, accounting for 0.5% of cases of congenital heart diseases.^1^ ALCAPA was first documented in a two-day old neonate by Konstantinowitsch in 1906.^2^ This anomaly is associated with early infant mortality or adult sudden death. Although it comprises a trivial part of congenital cardiac disease, its diagnosis is important and vital because of the high mortality rate registers. Among untreated cases, the mortality rate within the first year of life is about 90% secondary to different complications.^3-7^ In undiagnosed cases it leads to death in the fourth decade of life.^8-10^ Misdiagnosis of ALCAPA can be fatal, though early and correct diagnosis can be lifesaving. Here we present two misdiagnosed cases.

## Case presentation

### First case

The first case was a 3 year old female, weighing 12 kg, who had been diagnosed and managed as dilated cardiomyopathy since infancy. At the age of 12 months, she underwent diagnostic catheterization and was misdiagnosed as idiopathic dilated cardiomyopathy in spite of a dilated RCA. She was hospitalized for closure of a small patent ductus arteriosus (PDA) at the age of 36 months. Our physical examination revealed gallop rhythm and systolic regurgitant murmur of mitral valve. The electrocardiography (ECG) showed prominent Q wave in leads I, aVL, V5, and V6. Chest X-rays showed increased cardiothoracic ratio and prominent pulmonary vascular markings. Conventional transthoracic echocardiography examination showed severe dilation both in the left ventricle (LV) and left atria, moderate mitral regurgitation, low left ventricle ejection fraction (LVEF) 30%, dilated RCA and diastolic flow in main pulmonary artery and a small PDA.

Our findings after catheterization showed the following symptoms: increased oxygen saturation at pulmonary artery level up to 85% and elevated left ventricular end diastolic pressure (LVEDP) (Table 1).

**Table 1:** Cardiac Catheterization findings of the patients

		**Right atrium**	**Right ventricle**	**Main pulmonary artery**	**Left ventricle **	**Aorta**
Pressure, mmHg	First Case	Mean, 8	25/0-10	25/15, mean 20	130/0-20	130/80/110
	Second Case	Mean, 3	30/0-15	28/17, mean 23	105/0-20	100/70/85
Saturation	First Case	70%	70%	76%	95%	95%
	Second Case	70%	73%	77.5%	99%	99%

**Figure 1:**
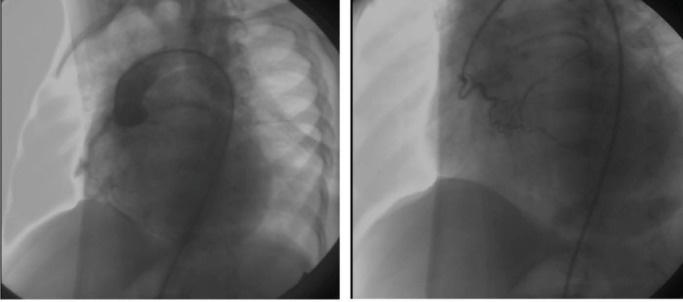
**left:** Filling of both coronary arteries simultaneously. **right:** Filling of LCA from conal branch

Aortic root and selective RCA injections revealed an almost simultaneous filling of both right and left coronary arteries. RCA was dilated and tortuous. Frame by frame cautious observation of the cine showed that the RCA filled short before the left coronary artery (LCA). This was due to the connection between the conal branch of the RCA and the LCA which was confirmed by selective injection at the conal branch of the RCA (Figure 1: right).

### Second case

**Figure 2:**
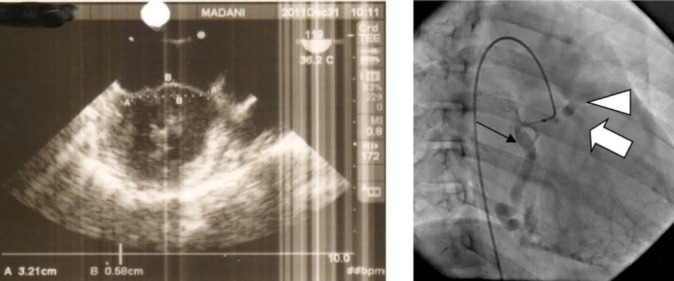
**left:** Transesophageal echocardiography shows dilated mitral valve annulus and prolapsed mitral valve leaflets about 6 mm. **right:** RCA (black arrow) injection at right anterior oblique (RAO) view shows opacification of left coronary artery LCA) (white arrow) and pulmonary artery (arrow head) consecutively.

The second case was a 10-year old female, weighing 33 kg, who had been followed by another medical center due to chest discomfort and diagnoses of prolapsed mitral valve. Her chest pain was aggravated by exertion. Physical examination revealed an apical click and second degree diastolic murmur at the upper left sternal border. ECG revealed ST Segment depression at lateral leads besides pathologic Q wave in lead aVL. The chest X-ray showed increased cardiothoracic ratio and pulmonary vascular markings. A conventional transthoracic echocardiography examination showed thickened and moderately prolapsed and insufficient mitral valve leaflets. (Figure 2: left). The most importantly detected sign was a severely dilated RCA which was as wide as 8 mm. There was also a diastolic blood flow from LCA toward the main pulmonary artery. Both the left atrium and LV were dilated. LV systolic function (EF=55%) was in about the normal range. The patient underwent catheterization leading to a diagnosis of the ALCAPA syndrome and prolapsed mitral valve. Cardiac catheterization showed increased LVEDP (about 20 mmHg) and 6% oxygen saturation step up at pulmonary artery level. Cardiac catheterization findings of both cases are summarized in Table 1.

Selective injection of RCA, showed consecutive opacification of LCA and main pulmonary artery (Figure 2: right).

## Discussion

ALCAPA was first described in an autopsy report as accessory arteries originating from the anterior PA to mediastinal and thoracic vessel like coronary arteries.^11^A high rate of sudden death has been reported in younger patients with ALCAPA and it is presumed that the mortality rate of ALCAPA declines after the fifth decade of life.^12^ Most articles underline the signs and symptoms of ALCAPA in infants and patients younger than 35 years of age who develop sudden death. In this report, we exhibited two cases with vague complaints presented in unusual stages of life. The first case was a three year old female who suffered from congestive heart failure. PDA was the main reason for which she was hospitalized. During our reassessment we became suspicious to ALCAPA. During an angiographic study, our frame by frame analysis of aortic root injection showed abnormal origination of the vessels. Demonstrating dilated RCA in conventional echocardiography was crucial and stressed that conventional transthoracic echocardiography may play an important role as the first diagnostic tool leading to a correct diagnosis of ALCAPA. We concluded that the dilated cardiomyopathy, diagnosed at the age of 2 years, was due to ischemic heart secondary to ALCAPA which was shown as deep Q waves in lateral leads of her ECG.

Our second case, a 10 year old female complained of chest pain which was thought to be due to her prolapsed mitral valve. Chest pain may present due to anxiety and other nonorganic causes. However, in this case as in the first, the critical clue was the pathologic ECG findings of ischemic heart and dilated RCA in conventional transthoracic echocardiography. On the contrary to the first case there was no clinical or lab findings of congestive heart failure.

Signs, symptoms, and findings of these two cases show that ALCAPA may present with vague signs and symptoms at different ages, ranging from a dull chest pain in a teenager to full blown clinical features, like severe dilated cardiomyopathy in early infancy. It is critical to remember that these vague and obscured clinical presentations of ALCAPA may be disclosed by ECG findings, careful transthoracic echocardiographic and selective aortic root and coronary artery angiographic studies with special attention to the RCA.

## Conclusion

It seems that ALCAPA is not a rare abnormality and correct diagnosis mostly depends on careful study and attention paid by physicians. Clinical presentation of ALCAPA in children may vary from sudden infant death to congestive heart failure beginning from infancy and a vague chest pain in teenage period.

## Ethical issues

The study was approved by the ethics committee of the University.

## Competing interests

Authors declare no conflict of interest in this study.
